# Comparison of Mercury Contamination in Live and Dead Dolphins from a Newly Described Species, *Tursiops australis*

**DOI:** 10.1371/journal.pone.0104887

**Published:** 2014-08-19

**Authors:** Alissa Monk, Kate Charlton-Robb, Saman Buddhadasa, Ross M. Thompson

**Affiliations:** 1 Institute for Applied Ecology, University of Canberra, Bruce, Australian Capital Territory, Australia; 2 School of Biological Sciences, Monash University, Clayton, Victoria, Australia; 3 Dolphin Research Institute, Hastings, Victoria, Australia; 4 Australian Marine Mammal Conservation Foundation, Hampton East, Victoria, Australia; 5 National Measurement Institute, Commonwealth Government, Port Melbourne, Victoria, Australia; Texas A&M University, United States of America

## Abstract

Globally it is estimated that up to 37% of all marine mammals are at a risk of extinction, due in particular to human impacts, including coastal pollution. Dolphins are known to be at risk from anthropogenic contaminants due to their longevity and high trophic position. While it is known that beach-cast animals are often high in contaminants, it has not been possible to determine whether levels may also be high in live animals from the same populations. In this paper we quantitatively assess mercury contamination in the two main populations of a newly described dolphin species from south eastern Australia, *Tursiops australis*. This species appear to be limited to coastal waters in close proximity to a major urban centre, and as such is likely to be vulnerable to anthropogenic pollution. For the first time, we were able to compare blubber mercury concentrations from biopsy samples of live individuals and necropsies of beach-cast animals and show that beach-cast animals were highly contaminated with mercury, at almost three times the levels found in live animals. Levels in live animals were also high, and are attributable to chronic low dose exposure to mercury from the dolphin's diet. Measurable levels of mercury were found in a number of important prey fish species. This illustrates the potential for low dose toxins in the environment to pass through marine food webs and potentially contribute to marine mammal deaths. This study demonstrates the potential use of blubber from biopsy samples to make inferences about the health of dolphins exposed to mercury.

## Introduction

Marine ecosystems are increasingly subject to a wide range of pressures, including over-exploitation, acidification, climate change, invasion and pollution [1]. Marine organisms in many areas are subject to high extinction risk as a result of human activities. Up to a third of coral reef fishes in northern Australia are considered vulnerable to extinction [2], and widespread extinctions are being observed across a range of marine taxa [1]. Marine mammals are considered especially vulnerable, and the International Union for the Conservation of Nature Red Book lists 25% of marine mammal species as threatened [3]. Recently a major analysis of the threatening processes for marine mammals identified those occurring in productive coastal waters as being particularly at risk, but also noted the lack of data on a number of rare species globally [4].

Coastal marine areas which are adjacent to major human population centres are subject to major threats as a result of human activities, including anthropogenic inputs of pollutants, particularly heavy metals [4], [5], [6]. The relative longevity of marine mammals, coupled with their high trophic position in food chains, results in potential for bioaccumulation of contaminants [7], [8]. One contaminant of particular concern is mercury. Mercury is highly toxic and has detrimental health effects in mammals including neurological disorders, immunosuppression and reproductive disorders that can all lead to death [9], [10]. Mercury is a naturally occurring element however centuries of human activities, including mining and coal burning, have led to increases in levels where it has become a major concern to both human and environmental health. In marine systems, mercury is retained in sediment where it can be taken up into the food web and biomagnified to high concentrations in the upper trophic levels and bioaccumulates in higher trophic organisms such as dolphins [11]. Mercury contamination in coastal waters therefore represents a potential major health risk to marine mammal populations.

Analysis of the factors contributing to marine mammal deaths is particularly problematic. Contaminant analysis of beach-cast dead animals at a number of locations internationally has found high levels of heavy metals in a range of taxa, including beluga whales [12], dolphins [13] and seals [14] (Table 1). However directly implicating anthropogenic contaminants in the deaths of marine mammals is difficult, because it is usually not possible to determine whether levels in dead animals are any higher than those in apparently healthy members of the population. It has been difficult therefore to assess the potential for heavy metals in the environment to directly impact on marine mammal mortality. This has contributed to the more general lack of data on the endangering processes which are impacting marine mammals [4].

**Table 1:** Comparison of concentrations of mean total mercury (mg/kg wet weight) found in beach-cast dead marine mammal populations worldwide.

Species	Area	Mercury		References
		*Blubber*	*Liver*	
*Tursiops australis*	Victoria, Australia	3.64	420.00	This Study
*Tursiops aduncus*	South Australia	-	475.78	[34]
*Tursiops truncatus*	Eastern Australia	-	16.36	[10]
*Delphinus delphis*	New Zealand	-	71.00	[8]
*Tursiops truncatus*	Israel coast, Mediterranean	1.50	97.00	[35]
*Stenella coeruleoalba*	Israel coast, Mediterranean	1.60	181.00	[35]
*Stenella coeruleoalba*	French coast, Mediterranean	0.86	217.73	[26] #
*Stenella coeruleoalba*	Apulian coast, Mediterranean	1.38	189.16	[36]
*Sousa chinensis*	Hong Kong	-	42.94	[9] #
*Phocoena phocoena*	England and Wales	-	16.15	[23]
*Stenella longirostris*	Gulf of California, USA	-	21.32	[37] #
*Tursiops truncatus*	South Carolina Coast, USA	-	17.8	[7]

# converted to wet weight using conversion factor of 1∶3 for liver and 1∶2 for blubber.

In 2011 a new species of dolphin, the Burrunan dolphin (*Tursiops australis*), was described from coastal waters of south-eastern Australia using a combination of genetic and morphological traits [15]. More recent phylogenetic analyses, using whole mitochondrial genome sequencing, further validates *T. australis* as a new species and a sister group to all other *Tursiops* lineages [16]. The species appears to be restricted to inshore waters of southern Australia (Victoria, Tasmania and South Australia [15], [17], [18], [19]. Only two small resident populations are known, from Port Phillip Bay and the Gippsland Lakes (approximately 100 and 50 individuals respectively. Both populations live in shallow semi-enclosed coastal water bodies which receive inflows from watersheds with extensive urban and agricultural development. In the case of the larger population (Port Phillip Bay) the animals live adjacent to Melbourne, a city of 4.5 million people. This has led to concern that the species may be subject to contamination from coastal pollution. This risk is intensified by evidence from population studies which have shown that population sizes of this species are small relative to other bottlenose populations in Australia and from around the world [15]. This species has also been proposed as a putative ancestral node for *Tursiops* diversification in this region [16] and as such is a highly significant species.

This study compares contaminant levels in beach cast and live *Tursiops australis* individuals to determine whether there is potential for contaminants to be contributing to dolphin mortality.

## Materials and Methods

### Ethics Statement

Collection of samples was conducted under the Wildlife Act 1975 Research Permit #10003250, issued by Victorian Department of Environment and Primary Industries (DEPI; Victorian State Government) and was approved by the Biological Sciences Animal Ethics Committee (Monash University) BSCI/2008/21.

We were able to assay contaminant levels (arsenic, lead, total mercury, selenium and summed polychlorinated biphenyls (PCBs)) in tissue from both beach-cast (‘dead’) and live *T. australis*. Ten blubber and six liver samples were obtained on necropsy from *T. australis* individuals found dead on beaches between 2004 and 2009. Tissue samples were collected during necropsies from fresh dead (code 2) or early moderate decomposition (code 3) carcasses (Table 2). Skin and blubber samples were taken from twenty live individuals (14 male and 6 female) using a dart biopsy approach [20] in 2007. Samples were stored at −20°C until analysed. The age of five of the dead animals was previously determined [19](Table 2). For live animals we limited our sampling to mature individuals to eliminate age class as a variable. Mature individuals were defined as being of a length of approximately 2.5 m.In addition, samples were taken from fish species in the region which are known to form part of the dolphins' diet [19], in order to determine likely pathways for mercury ingestion. Mercury levels were measured in the main food web compartments through sampling of 5–10 g of muscle tissue from fish. Fish were sourced from local fishermen who operate in the same areas as the dolphins feed.

**Table 2:** Biological characteristics of beach cast individuals of *Tursiops australis* from Victoria, Australia.

Location	Date collected	Sex	Age (yrs)	Length (m)	Decomposition code	Blubber layer at dorsal fin (cm)
Altona	02/10/2004	F	-	-	3	.5
Geelong	19/09/2005	F	-	2.62	2	2.5
Port Fairy	27/10/2006	M	8	2.66	3	1.5
San Remo	23/04/2007	M	-	2.27	3	1.5
Poddy Bay	25/10/2007	M	11	2.7	3	-
Mitchell River	01/11/2007	F	20	2.78	3	1.8
Paynesville	04/11/2007	M	-	2.73	2	1.5
Beaumaris	21/01/2008	M	21	2.55	3	1.2
Point Henry	23/01/2008	M	13	2.36	2	1.4
Clifton Springs	14/11/2008	M	-	2.20	2	1.4

Sample preparation and subsequent toxicological analysis of both dolphin and fish tissue was carried out at the National Measurement Institute (NMI) (Melbourne, Victoria; Commonwealth Government). Contaminant levels were determined on fresh tissue using a standard Nitric Acid digestion and detection with both ICP-MS and ICP-OES. Certified Reference Materials were analysed together with the samples for quality assurance. To validate whether using biopsy sized blubber portions (weight range 2–3 g) was comparable to larger samples (50–100 g) taken from necropsied animals, biopsy-sized sub-samples were taken from the larger samples and mercury results compared between the two methods (all other contaminants occurred at very low levels, Table 3). No significant differences were found (F_(1,14)_ = 0.17, p = 0.69). We also tested whether there was any difference in mercury levels based on gender and there were no significant sex differences in mercury concentration for either live (F_(1,18)_ =  0.62, p = 0.40) or beach cast dolphins (F_(1,8)_ = 0.32, p = 0.81), therefore sex was pooled for all further analyses. We investigated whether there was a temporal effect on mercury levels in beach cast dolphins and no effect was found (F_(1,8)_ = 0.107, p = 0.752).

**Table 3:** Concentrations of contaminants (mg/kg wet weight) in blubber from individuals of *Tursiops australis* from Victoria, Australia.

*Contaminant*	Dead(n = 10)	Live (n = 20)
**Arsenic**	0.39±0.07	0.23±0.02
	(0.13–0.80)	(<0.10–0.38)
**Lead**	0.061±0.011	2.91±0.615
	(0.05–0.14)	(0.76–12)
**Total Mercury**	3.64±0.68	1.32±0.20
	(1.40–7.20)	(0.32–4.20)
**Selenium**	2.88±0.74	1.69±0.2
	(0.80–6.50)	(0.52–3.9)
**∑PCB**	3275.54±9.46.28	-
	(258.80–8055.3)	
		

Values shown are means with standard errors and range in brackets underneath.

As most published studies have reported mercury levels in liver tissue, which we could not sample in live dolphins, we combined data from this study and data from previous published studies (see studies with both blubber and liver mercury concentrations Table 1) to form a regression between liver and blubber levels, (n = 30 R^2^ = 0.59, p<0.001, ln(liver level) = 1.1107× ln(blubber level)+4.5851) and converted values from live dolphin blubber samples to liver concentrations to facilitate comparison with previously published studies.

## Results and Discussion

Arsenic, lead, selenium and PCBs were detected but were lower than levels known to cause health effects [21] (Table 3). We acknowledge that it is likely a combination of contaminants that burden the animals, however given that no other contaminant tested is at high levels it is likely that mercury is contributing the majority of the toxicological burden on these animals.

**Figure 1:**
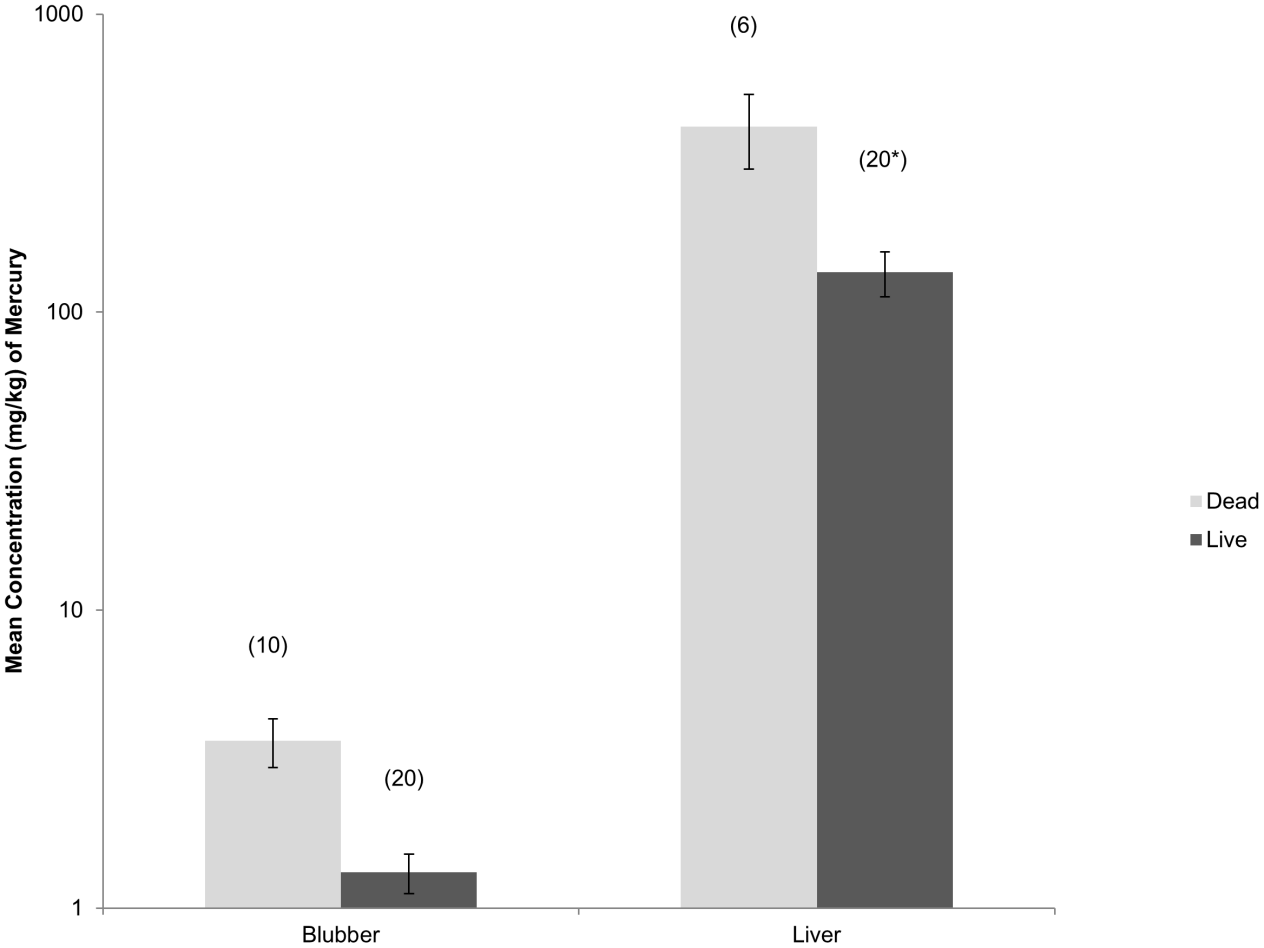
Concentration of total mercury (mg/kg wet weight) in blubber and liver from live and dead individuals of *Tursiops australis* from Victoria, Australia. Values shown are means with standard errors, sample sizes are shown in brackets above each bar. Values marked * were estimated from a regression between liver and blubber levels from worldwide levels.

The total mercury in blubber of dead adult *T. australis* from coastal Victoria was 2.7 times higher on average than the values for the live animals (Figure 1) (one factor ANOVA for a comparison between live and dead animals; F_(1,28)_ = 36.04, p<0.001). For the beach-cast animals, liver values ranged from 100 to 840 mg/kg, while in live animals the range was estimated to be between 28 and 483 mg/kg. Placed in an international context, *T. australis* has a higher average concentration of mercury than has been reported for small cetaceans in East Australia, New Zealand, America, England, Hong Kong and the Mediterranean (Table 1).

There is little information on actual physiological tolerances to contaminants in free-living animals, however it has been noted that there is a limit to the concentration of mercury that an animal can tolerate. For mammalian liver tissues, this limit appears to be within the range of 100–400 mg/kg, wet weight, of liver tissue [21]. In study by Ronald *et al.*, [22], seals were fed different daily doses of mercury. Compared to the control group, the low dose group showed a decline in appetite and body weight and a reduction in activity after approximately 60 days. The high dose group had to be force fed after 4 days, became lethargic, suffered from continuous weight loss and had died by day 26. This shows that even at lower doses, mercury is having behavioural effects and at high doses it can cause death. The average total mercury concentration in the liver of the high dose animals after death was 138 mg/kg. Nearly all dead dolphins from this study had higher mercury levels in their livers than the dead seals from the Ronald *et al.*, [22] study. It is also important to note that some of the predicted liver concentrations of live (assumed healthy) dolphins from this study also exceeded this concentration, emphasising the importance of knowing the mercury concentrations in the live populations before making any conclusions about the possible effects in dead animals. Bennett *et al.*, [23] found that harbor porpoises that died from infectious disease had significantly higher mercury levels than healthy animals that died from physical trauma, suggesting that mercury causes immunosuppression at levels much lower than found in the current study (Table 1). Full histopathology studies were not carried out in this study and therefore a relationship between the presence of infectious disease and mercury concentrations could not be determined. Studies into the neurological effects of mercury in seals [24] and polar bears [25] have found that low levels of mercury affects neurochemical pathways that have essential roles in multiple aspects of animal health, behaviour, reproduction, and survival. Based on these studies and given the high concentration of mercury found in *T. australis*, it is highly likely that mercury is affecting animal health, potentially through immunosuppression and influences on neurochemical pathways.

Ratios of mercury: selenium in liver tissue are indicative of toxicological stress in mammals as physiological processes act to bind methyl mercury and store it as the insoluble compound tiemmannite (HgSe) [26], [27]. This detoxification process results in a 1∶1 molar ratio of mercury and selenium. An average ratio of 1∶1.06±0.03 was found in the beach-cast Victorian dolphins, suggesting these dolphins are under toxicological stress from mercury.

This study represents a significant advance by being able to compare beach-cast to live animals within the same population, potentially implicating mercury in the morbidity of animals within a region. The ability to biopsy live animals and compare blubber mercury concentrations to those from beach-cast individuals has considerable potential in marine mammal studies. Whilst a previous study by Stavros *et al.*, [28] has found a slightly stronger relationship between mercury levels found in liver and skin, and used the method to make inferences about the health of free ranging animals, there are some limitations to using their method. Their study involved capture and release of animals and as such they are able to obtain larger samples of skin (approximately 0.5 g dry weight) than is able to be collected from the dart biopsy approach (0.03 g wet weight). Skin samples from our study were small and used for genetic analyses preferentially over toxicological analyses, meaning that the use of the blubber component was the only practical approach.

Age has been found to be an important factor in mercury concentrations in marine mammals. As an animal gets older, the level of mercury is predicted to increase due to bioaccumulation [29]. Given only five animals in this study were aged it was not realistic to determine whether there was any association between mercury contamination and age. We can however state that the individuals sampled were not all older animals and, importantly, that levels observed were not highest in the oldest animals. We can conclude that the animals dying are not just old age animals that have accumulated high levels of mercury over their long lifetime, as three out of the four animals aged were considered middle aged (Table 2).

**Figure 2:**
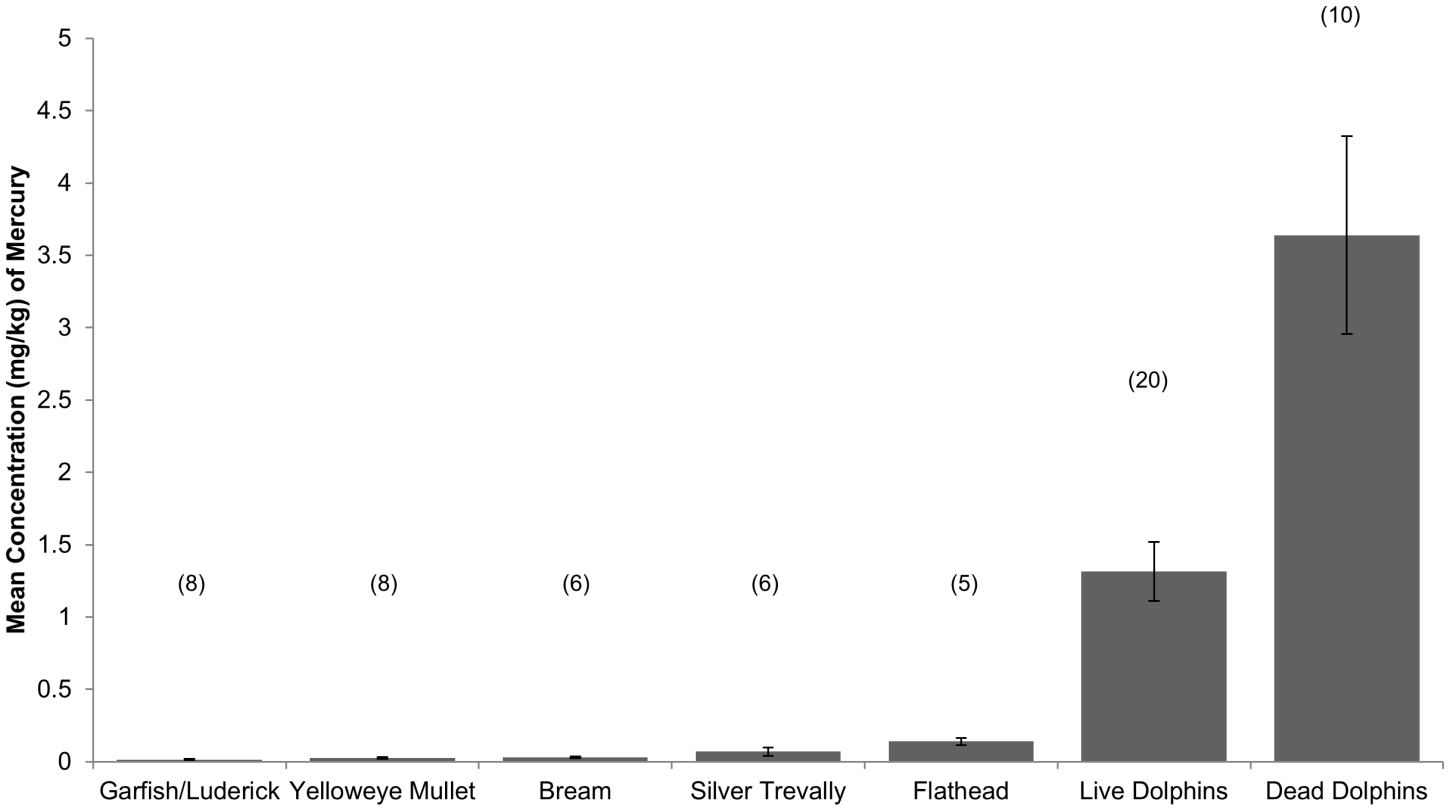
Concentration of total mercury (mg/kg wet weight) in potential dolphin prey muscle and blubber from live and dead *Tursiops australis* from coastal Victoria. Values shown are means with standard errors, sample sizes are shown in brackets above each bar.

We were not able to clearly identify a single point of origin for the mercury. Of note, the region was extensively mined for gold in the late 19^th^ and early 20^th^ Centuries, activities which included wide spread use of mercury to extract gold ore [30]. It is likely that mercury is entering the semi-enclosed waters in which *T. australis* is found via major river catchments and becoming incorporated into the marine food web. Using a previous stable isotope study of trophic relationships for this species [19] we were able to identify the main prey species of the dolphins and assess their mercury levels. Analysis of mercury loads in dolphin prey found no clear single source of contamination, although all prey items contained measurable amounts of mercury (Figure 2). Mercury is readily absorbed across the gastro-intestinal tract from prey items, and the volumes of food ingested over the lifetime of an animal mean that even low doses in prey items may result in high levels of exposure [29]. The levels of mercury found in prey tissue were much lower than those in the dolphins, consistent with a number of previous studies of marine mammals [29], [31]–[33]. However mercury is strongly biomagnified in marine food chains [31]–[33], and bioaccumulates in muscle tissue, blubber and in the liver of marine mammals [29]. The values that we found indicate that even relatively low doses of contaminants in marine environments can result in high loadings to long-lived, high trophic level predators. A comprehensive food web study is needed to be able to calculate the amount of mercury *T. australis* is consuming.

Our comparison of levels from beach-cast and live animals shows the potential for a role for mercury contamination in the mortality of these animals. This, along with the fact that mercury concentrations were within a range known to have multiple health effects, would suggest that mercury is negatively impacting on these dolphins. This is of grave concern, considering that this species is newly described, appears to occur over a very limited range and has a small known population size. However a limitation of this study is that we only analysed for total mercury. We acknowledge that to better understand the effects of mercury, speciation of mercury should be completed to identify what form the mercury is in. Another limitation of the study is that we didn't do a screen for all possible contaminants, in particular dioxins, PAHs and organic pesticides that are known to have detrimental effects at low concentrations. Pilot work on dolphins from this area has suggested that concentrations of these contaminants are low (Monk, unpublished data) but we cannot state with certainty that they are not contributing to mortality in some cases.

To better understand and interpret the levels present in dead dolphins from a population, it is important to compare it to levels found in the live population. Future toxicological research should also include parallel studies to investigate the actual effects of mercury on the dolphins including histopathology, measurement of neurochemical biomarkers and measurement of genomic and genetic biomarkers.
