# Correction: Comparison of Mercury Contamination in Live and Dead Dolphins from a Newly Described Species, *Tursiops australis*

**DOI:** 10.1371/journal.pone.0113861

**Published:** 2014-11-12

**Authors:** The PLOS ONE Staff

The legend for Table 3 should note that ∑PCB concentrations are µg/kg wet weight. Please see the complete, corrected Table 3 here.

**Table 3:** Concentrations of contaminants (mg/kg wet weight, ∑PCB concentrations are µg/kg wet weight) in blubber from individuals of *Tursiops australis* from Victoria, Australia.

*Contaminant*	Dead(n = 10)	Live (n = 20)
** Arsenic**	0.39±0.07	0.23±0.02
	(0.13-0.80)	(<0.10-0.38)
** Lead**	0.061±0.011	2.91±0.615
	(0.05-0.14)	(0.76-12)
** Total Mercury**	3.64±0.68	1.32±0.20
	(1.40-7.20)	(0.32-4.20)
** Selenium**	2.88±0.74	1.69±0.2
	(0.80-6.50)	(0.52-3.9)
** ∑PCB**	3275.54±9.46.28	-
	(258.80-8055.3)	

Values shown are means with standard errors and range in brackets underneath.

## References

[1] Monk A, Charlton-Robb K, Buddhadasa S, Thompson RM (2014) Comparison of Mercury Contamination in Live and Dead Dolphins from a Newly Described Species,*Tursiops australis* . PLoS ONE 9(8): e104887 doi:10.1371/journal.pone.0104887 25137255 10.1371/journal.pone.0104887 PMC4138083

